# Bayesian Youden index for algorithmic evaluation under class imbalance: mathematical foundations with applications to insulin resistance and diabetes progression

**DOI:** 10.3389/fepid.2026.1839151

**Published:** 2026-08-26

**Authors:** Aquiles Darghan, Ariel Iván Ruiz-Parra, Jorge Eduardo Caminos, Nair González, Kevin Darghan, Sofia Alexandra Caminos Cepeda

**Affiliations:** 1 Faculty of Agricultural Sciences, Universidad Nacional de Colombia, Bogotá, Colombia; 2 Faculty of Medicine, Universidad Nacional de Colombia, Bogotá, Colombia; 3 Faculty of Engineering, Universidad Nacional de Colombia, Bogotá, Colombia; 4 Data Science Engineering, Universidad EAN, Bogotá, Colombia; 5 School of Medicine, Universidad Pompeu Fabra, Barcelona, Spain

**Keywords:** HOMA-IR, negative predictive value, positive predictive value, sensitivity, specificity

## Abstract

**Background:**

The evaluation of binary classifiers under real-world conditions requires metrics that account for the dependence of predictive performance on disease prevalence. Classical accuracy metrics, including the Youden index, condition on the true class and remain invariant to changes in class distribution, making them unable to reflect the practical question of whether a positive classification is trustworthy in a given deployment context.

**Methods:**

The Bayesian Youden Index is derived analytically as a prevalence-dependent extension of the classical Youden index, grounded in the equivalence between Bayesian and classical sensitivity and specificity. Its behavior is characterized across the full prevalence range, and uncertainty in all point estimates is quantified through parametric bootstrap with B = 2,000 replications. The framework is illustrated using HOMA-IR for insulin resistance detection (*n* = 93) and is applicable to any binary classification task with a known confusion matrix and operational prevalence.

**Results:**

The Bayesian Youden Index reaches a maximum of 0.913 (95% CI:0.823,0.997) at a prevalence of 0.334 (95% CI:0.027,0.575), compared to the constant classical value of 0.890 (95% CI:0.782,0.978). At the observed prevalence of 0.484 the Bayesian value is 0.898. Two intersection points were identified at which the Bayesian and classical formulations coincide: $P_{1}=0.190$ (95% CI:0.118,0.542) and $P_{2}=0518$ (95% CI:0.504,0.652), defining three distinct prevalence regimes with different implications for classifier deployment. The index exhibits asymmetric behavior across the prevalence range, showing that a classifier's inherent sensitivity-specificity profile can be strategically leveraged through prevalence-aware threshold selection to manage class imbalance without resorting to artificial balancing techniques.

**Conclusions:**

The Bayesian Youden Index positions itself not merely as a measure of statistical fit but as a robust calibration tool that decomposes overall classifier performance into predictive confidence under realistic prevalence conditions. The findings caution against prematurely discarding results from imbalanced datasets or automatically applying balancing methods, advocating instead for a nuanced analysis in which imbalance, mediated by classifier characteristics, can sometimes enhance rather than hinder evaluated performance.

## Introduction

The evaluation of diagnostic tests remains of interest in medicine and the biological sciences, as it elucidates the operational characteristics of new diagnostic tools, whether by comparison with an established standard or for validation purposes. A complete understanding of the measures used can help curb misuse of resources and avoid unnecessary testing, while enabling new diagnoses, therapies, and outcome predictions. The discriminative ability of a test is largely influenced by its design and purpose, as well as the type of data produced. For continuous data, normal and abnormal results are discriminated by differences in mean values or by a cutoff point (1). For binary data, as in the case of insulin resistance classification, this may involve a supervised approach where the label is available because a gold standard such as the Hyperinsulinemic-Euglycemic Clamp has been used to create the dichotomy (2), or validation through genetic analysis of attributes associated with a disease (3).

Usually, the decision to define a cutoff point for a binary classifier based on surrogate indices to assess insulin sensitivity can be somewhat challenging, particularly when testing a new technology. A surrogate index, in this context, is an algebraic formula computed from routinely available, minimally invasive measurements, typically fasting glucose and insulin, used as a proxy for a reference procedure such as the hyperinsulinemic-euglycemic clamp or an oral-glucose-tolerance-test-based index, which are too invasive, time-consuming, or costly for routine screening. Because these formulas are not derived from a mechanistic model of insulin action but are empirical approximations, they lack a universal physiological cutoff; a decision threshold must instead be calibrated against a reference method within each population, which is why reported cutoffs vary appreciably across studies and cohorts (4, 5). This process typically requires an exhaustive literature review, expert or feasibility opinions, as well as pilot studies. Once the cutoff point has been established in this manner, or alternatively through an algorithm that determines the value by optimizing an objective function until convergence, it is possible to obtain the sensitivity and specificity measures of the test. These measures are ubiquitous in diagnostic testing and algorithmic performance evaluation, as well as other related metrics, such as the Youden index (5). Other characteristics of diagnostic tests, such as positive and negative predictive values, are often explored together when sensitivity and specificity are evaluated. However, they are typically considered complementary measures without the recognition that, in essence, they represent sensitivity and specificity from a Bayesian perspective. This Bayesian framework can incorporate patient-specific or population-level characteristics to estimate prior probabilities, conceptualized as pre-test disease likelihood in clinical diagnostics or as baseline class prevalence (as a form of natural partitioning of classes) in algorithmic classification. In diagnostic settings, these priors inform test selection and result interpretation; in algorithmic contexts, they influence threshold calibration and model deployment decisions across populations with varying prevalence rates (unbalanced classes). The posterior probability updated via observed outcomes serves the same fundamental purpose in both frameworks: transforming uncertain priors into actionable predictions. This dual applicability is particularly relevant when observed binary labels are processed through classification algorithms to evaluate predictive performance.

Independence between gold standard measurements and diagnostic tests is a foundational assumption in test validation, as violations introduce systematic biases that compromise performance estimates (4). This principle applies to insulin sensitivity assessment, where reference methods establish classification benchmarks. Certain indices provide well-defined insulin resistance thresholds, whereas surrogate indices such as QUICKI, HOMA-IR, and METS-IR exhibit less clear decision boundaries (6). However, when a surrogate index is optimized against a well-validated benchmark, this calibration approach can reduce misclassification rates while preserving the surrogate's practical advantages and enhancing its generalizability across populations without merely replicating the reference method.

The present study integrates classical and Bayesian frameworks for estimating sensitivity and specificity to evaluate insulin resistance classification performance, extending the conventional Youden index optimization approach. While Bayesian methods have been applied across diverse domains (7, 8), their application to insulin resistance surrogate index validation remains underexplored. We illustrate this methodology using HOMA-IR as the primary exemplar, with results also reported for QUICKI, Matsuda, METS-IR, and TyG. QUICKI and HOMA-IR require only fasting glucose and insulin; the Matsuda index requires a full oral glucose tolerance test and serves as the reference standard in the source dataset; METS-IR and TyG are indirect indices that substitute anthropometric and lipid measures for a direct insulin value. This set was selected to span the practical range from the simplest fasting-only indices to the Oral Glucose Tolerance Test-based reference index (9), allowing the framework to be exercised across indices with different operating characteristics rather than a single case. Binary classification performance is evaluated through the algorithmic framework detailed in (6), enabling systematic comparison of discriminatory capacity and optimal threshold determination across indices. This approach integrates standard diagnostic tools with Bayesian reasoning, enabling comparison of classification results across indices and examination of the relationship between classical and Bayesian sensitivity and specificity. Furthermore, it extends the Youden index by incorporating predictive values, revealing a connection with the eigenvalue structure of the confusion matrix that is not merely analogical but emerges from a mathematically sound foundation linking the structural components of classification with their probabilistic interpretation.

The Bayesian framework provides a threshold-optimization framework that accommodates moderate class imbalance through its probabilistic formulation, offering a transparent alternative to data-level augmentation techniques when the imbalance is not severe enough to warrant the additional complexity and assumptions that such techniques introduce. This threshold-level correction is not an isolated technical convenience but an expression of a deeper integrative logic that runs through the entire framework. This integrated framework yields several methodological advances. First, it enables direct comparison of classification performance across insulin resistance indices through both classical and Bayesian lenses. Second, it demonstrates the mathematical equivalence between Bayesian sensitivity/specificity and positive/negative predictive values, clarifying their conceptual relationship. Third, it extends the Youden index to incorporate prevalence through Bayesian formulations, explicitly addressing class imbalance, a critical consideration in machine learning applications (10). Finally, eigenvalue analysis of the confusion matrix reveals an interesting relationship between its extremes and the extended Bayesian formulation of the Youden index, demonstrating that this extension is not merely analogical but is mathematically grounded in the underlying structure of classification. This prevalence-dependent formulation proves particularly valuable for evaluating algorithmic fairness across demographic subgroups with differing insulin resistance rates.

Although the present study develops and validates the Bayesian Youden index within the insulin resistance domain, the index is not domain-specific. Its sole inputs are the four cells of any confusion matrix and the class prevalence, quantities universally available across binary classification problems regardless of clinical context, dataset origin, or degree of imbalance. This means that any investigator reporting a confusion matrix in any domain can compute Bayesian Youden index immediately, without additional data collection, model retraining, or algorithmic intervention. The portability of the index was confirmed by applying it to published confusion matrices from an independent study in a clinically distinct setting (11), where the classical Youden index failed to reflect the differential impact of class prevalence across tasks that Bayesian Youden index captured transparently. This external illustration confirms that the sensitivity of Bayesian Youden index to prevalence is not an artifact of the insulin resistance setting but a structural property of the index that generalizes to any binary classification context where the distribution of classes carries meaningful information about real-world performance.

## Classical and Bayesian approaches

In the frequentist framework, an unknown quantity, for instance, the population probability that a subject has insulin resistance is treated as a fixed but unknown constant. Probability statements attach to the sampling procedure rather than to the parameter itself: were the study repeated many times under identical conditions, a given confidence interval would contain the true value in a specified proportion of repetitions. This repeated-sampling justification underlies inferential statements such as confidence intervals and hypothesis tests, but is not required for purely descriptive quantities, such as the sample sensitivity or specificity computed directly from an observed confusion matrix.

In the Bayesian framework, by contrast, the unknown parameter is itself treated as a random variable. A prior distribution encodes what is believed about the parameter before the data are observed; the likelihood describes how probable the observed data are under each candidate value of the parameter; and Bayes' theorem combines the two into a posterior distribution, the updated state of belief after observing the data. Point estimates and interval estimates follow directly from this posterior, rather than from hypothetical repetition of the experiment. Under this view, the probability that a given patient has insulin resistance is not an underlying frequency but a degree of belief, on a scale from 0 (certainty of absence) to 1 (certainty of presence), that is coherently revised as new data, a new likelihood is incorporated into the prior (12).

In clinical studies, it is common for included patients not to come from a strict probability sample, but rather from accessible populations such as those attending a hospital or meeting specific inclusion criteria. In this context, selection bias affects the external validity of diagnostic tests, as the sensitivity and specificity values may not reflect the test's performance in the general population. However, when discussing algorithmic performance, the emphasis shifts, as what becomes relevant is the representativeness and balance of the data used to train and validate the model. Thus, sensitivity and specificity remain useful metrics because they describe the proportion of true positives and true negatives within the analyzed dataset, but their extrapolation depends more on the quality of the dataset than on the clinical selection of patients. In summary, while in diagnostic tests selection bias limits generalizability, in algorithms the challenge lies in avoiding data biases to ensure that the observed performance can be transferred to other populations or scenarios (12, 13).

## Sensitivity and specificity in algorithmic performance testing

The following briefly describes the characteristics of sensitivity and specificity used in evaluating the performance of classification algorithms. In binary classification tasks, sensitivity, or the true positive rate (*TPR*), is defined as the proportion of instances with the positive class label that are correctly identified by the algorithm. This is complementary to the false negative rate (*FNR*). Specificity, or the true negative rate (*TNR*), is defined as the proportion of instances with the negative class label that are correctly classified as such by the algorithm and is complementary to the false positive rate (*FPR*) (14). It is important to note that the designation of which class is considered “positive” or “negative” is arbitrary and context-dependent, either class can be labeled as positive depending on the research question or practical application. Both measures, commonly applied to diagnostic tests, have been extended to algorithm performance evaluation, where true positives (TP), false positives (FP), true negatives (TN), and false negatives (FN) arise when comparing the algorithm's binary classifications with the observed or ground truth labels from the data.

All occurrences can be summarized in a 2 × 2 confusion matrix as shown in Table 1, illustrated with an example related to insulin resistance (IR), for which several non-invasive detection, diagnosis, and monitoring methods exist. The data for this example come from an exploratory cross-sectional study with binary labels: IR (insulin resistance present) and NIR (no insulin resistance). In general terms, the confusion matrix relates the cause to the observed values (columns in Table 1), denoted as $y=IR_{O}$ or $y=NIR_{O}$, and the rows to the effect or values classified by the algorithm, denoted as $\hat{y}=IR_{A}$, $\hat{y}=NIR_{A}$, with $n(IR_{O})=n(TP)+n(FN)$, $n(NIR_{O})=(FP)+n(TN)$, $n(IR_{A})=n(TP)+n(FP)$ and $(NIR_{A})=$ n(FN)+n(TN).

**Table 1:** Confusion matrix for the binary classifier.

		Cause: Observed Value		Sub-total
		$y=IR_{O}$	$y=NIR_{O}$	
Value Classified by the Algorithm	$\hat{y}=IR_{A}$	n(TP)	n(FP)	$n(IR_{A})$
	$\hat{y}=NIR_{A}$	n(FN)	n(TN)	$n(NIR_{A})$
Sub-total		$n(IR_{O})$	$n(NIR_{O})$	n

**Figure 1:**
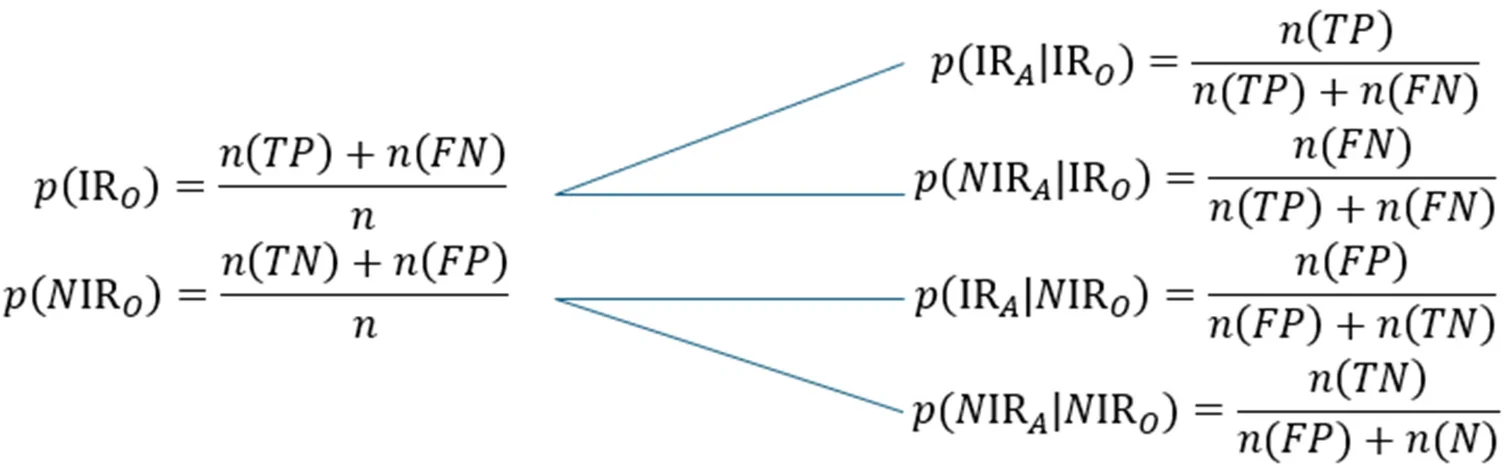
Tree diagram and the probability distribution of each event (*A*, algorithm and *O*, observed). The notation n(.) represents the count of each event.

To better understand the calculation of both classical and Bayesian sensitivity, the tabular information is represented in a tree diagram (Figure 1). Probabilities are assigned based on the counts for the events in Table 1. Here, $p(IR_{O})$ represents the probability that a subject is insulin-sensitive in the observed data (the prior probability). Its complement is $p(NIR_{O})$ where $\left(p(NIR_{O})\;=\;1\text{-}p(IR_{O})\right)$, $p(IR_{A}|IR_{O})$ is the conditional probability that, given a subject is insulin-sensitive in the observed data, they are classified as insulin-sensitive by the applied algorithm, with $p(NIR_{A}|IR_{O})$ as its complement. Finally, $p(IR_{A}|NIR_{O})$ is the conditional probability that, given a subject is not insulin-sensitive in the observed data, they are classified as insulin-sensitive by the applied algorithm, with $p(NIR_{A}|NIR_{O})$ as its complement.

A classification algorithm should have the following properties: a high count of true positives or sensitivity. If the algorithm produces a poor classification, it should generate an alert for concern. Sensitivity is estimated in a classical manner as the conditional probability $p(IR_{A}|IR_{O})$. Similarly, if there is a high rate of true negatives, there is no need for concern if a subject who is *N*IR, is classified as such by the algorithm in most iterations (in an iterative scenario). Specificity is estimated as the conditional probability $p(NIR_{A}\;|\;NIR_{0})$, which can also be interpreted as a low false positive rate or a lower frequency of classifications as IR*_A_* given that the subject was $NIR_{o}$ (13). Using the cell counts from the contingency table in Table 1, the classical sensitivity $\left(C_{se}\right)$ is defined as:

$$C_{se}=p(IR_{A}|IR_{O})=\frac{n(TP)}{n(TP)+n(FN)}=\frac{n(TP)}{n(IR_{O})}$$ (1)

and the specificity $\left(C_{se}\right)$ as:

$$C_{sp}=p(NIR_{A}|NIR_{O})=\frac{n(TN)}{n(TN)+n(FP)}=\frac{n(TN)}{n(NIR_{O})}$$ (2)

In this sense, an algorithm with good classification performance is not “opposite” to Bayesian indices but is evaluated from a complementary perspective: while sensitivity (Equation 1)and specificity (Equation 2) describe intrinsic properties of the algorithm, Bayesian indices incorporate prior probability and allow for the estimation of predictive values. Both approaches aim to determine whether a medical condition (or outcome) exists, but they do so from distinct and complementary viewpoints. While classical sensitivity and specificity describe the algorithm's intrinsic properties relative to the true state of the condition, the Bayesian approach answers a different question: What is the probability that the true state corresponds to what the algorithm predicted? In this sense, Bayesian sensitivity $p(IR_{O}|IR_{A})$ and Bayesian specificity $p(NIR_{O}|NIR_{A})$ shift the analysis from the internal performance of the algorithm toward the clinical interpretation of its results, additionally integrating the prevalence of the condition in the population. It is worth stating from the outset that Bayesian sensitivity and Bayesian specificity, once derived, are algebraically identical to the predictive values, quantities already standard in diagnostic testing and machine learning evaluation. The derivation that follows is presented not to introduce new measures, but to make explicit, via Bayes' theorem, why predictive values are properly understood as the Bayesian counterparts of sensitivity and specificity; this equivalence is what motivates the Bayesian extension of the Youden index developed in the next section.

When applying Bayes' theorem to calculate these probabilities, we can associate the corresponding quantities with Bayesian sensitivity $\left(B_{se}\right)$ and Bayesian specificity $\left(B_{sp}\right)$ respectively as:

$$B_{se}=p(IR_{O}\;|IR_{A})=\frac{\;p(IR_{O})p(IR_{A}\;|IR_{O})}{\;p(IR_{A})}$$

which, when developing $p(IR_{A})$, yields:

$$\;B_{se}=\frac{\;p(IR_{O})p(IR_{A}\;|IR_{O})}{\;p(IR_{A}\;|IR_{O})p(IR_{O})+p(IR_{A}\;|NIR_{O})p(NIR_{O})}$$ (3)

From Equation 3 and using the usual definition of classical sensitivity $\left(C_{se}\right)$, the expression given in Equation 4 can be obtained through simple calculations:

$$B_{se}=\frac{(n(TP)+n(FN))C_{se}}{\left(n(TP)+n(FN))C_{se}+n(FP\right)}=\frac{n(TP)}{n(IR_{A})}$$ (4)

Thus, $B_{se}$ (Equation 4) is simply the proportion of true positives conditioned on the set of cases that the algorithm classifies as $IR_{A}$, which aligns with the Bayesian approach, the straightforward posterior probability of the *TP*. Proceeding similarly for specificity yields:

$$\;B_{sp}=p(NIR_{O}|NIR_{A})=\frac{\;p(NIR_{O})p(NIR_{A}\;|NIR_{O})}{\;p(NIR_{A})}$$ (5)

Developing $p(NIR_{A})$ and simplifying it using the standard expression from the classical approach for $\left(C_{sp}\right)$ yields:

$$\;B_{sp}=\frac{(n(TN)+n(FP))C_{sp}}{\left(n(TN)+n(FP))C_{sp}+n(FN\right)}=\frac{n(TN)}{n(NIR_{A})}$$ (6)

Thus, $B_{sp}$ (Equation 5) is the proportion of true negatives conditioned on the set of cases that the algorithm classifies as $NIR_{A}$, which was intuitively expected from the previous result, namely, the posterior probability of the *TN* event.

As can be observed from Equations 4, 6, these expressions correspond to Positive Predictive Value (*PPV*) and Negative Predictive Value (*NPV*), respectively. Consequently, studies evaluating classification performance through predictive values are implicitly adopting a Bayesian perspective, a connection often overlooked in algorithmic contexts (15). Recognizing *PPV* and *NPV* as “Bayesian sensitivity” and “Bayesian specificity” clarifies that these metrics inherently integrate prior information (class prevalence) with classifier discriminatory capacity. Unlike classical sensitivity and specificity, which remain invariant to class distribution, predictive values depend critically on prevalence. This distinction has profound implications for machine learning: a classifier with high sensitivity may exhibit low *PPV* when deployed in populations where the positive class is rare, necessitating prevalence-aware threshold calibration and stratified performance evaluation across demographic subgroups with varying base rates.

## Bayesian extension of the Youden Index

Building on these results, standard performance metrics can be readily extended to the Bayesian framework. Before presenting the extension, it is worth motivating why a single prevalence-dependent index, built on the same additive structure as the classical Youden index, is preferable to reporting the positive and negative predictive values as a separate pair. A single summary statistic of this form remains directly comparable, number for number, to the classical Youden index already used throughout the diagnostic-testing literature, in a way that two separately reported predictive values do not. It also allows a classifier's overall balance between predictive confidence in a positive result and predictive confidence in a negative result to be read from a single value at a given prevalence, rather than requiring the two predictive values to be interpreted jointly and informally. This motivates constructing the Bayesian analogue of the classical Youden index by replacing sensitivity and specificity with their Bayesian counterparts in the same expression:

Two commonly used measures are the Youden index (16) and test accuracy (17). The classical Youden index is defined as:

$$C_{Y}=C_{se}+C_{sp}\text{-}1=\frac{n(TP)}{n(IR_{O})}-\frac{n(FP)}{n(NIR_{O})}$$ (7)

Thus, for the Bayesian case it would be:

$$B_{Y}=\frac{n(TP)}{n(IR_{A})}+\frac{n(TN)}{n(NIR_{A})}\text{-}1=\frac{n(TP)}{n(IR_{A})}-\frac{n(FN)}{n(NIR_{A})}$$ (8)

In Equation 8 connects sensitivity and specificity with posterior probabilities (predictive values), demonstrating how the Bayesian approach transforms the Youden index into a tool more aligned with clinical practice, where what matters is the probability of a disease, condition, or outcome given a result. In the algorithmic domain, this same formulation can be interpreted to translate classical metrics into a probabilistic framework, integrating prevalence (Partitions — from an algorithmic classification perspective) and context into the evaluation of a model's performance.

The mathematical analogy between the classical Youden index $C_{Y}$ (Equation 7) and a similar construction based on predictive values gives rise to an alternative metric whose domain is identical to that of the original index. While $C_{Y}$ summarizes the intrinsic discriminative ability of a test, the predictive-value version provides a global performance measure that incorporates the influence of prevalence, thus offering an interpretable and useful synthesis in applied contexts. This extension should not be regarded as a mere *ad hoc* variation, but rather as a coherent formulation that transfers the mathematical structure into a predictive framework, broadening the scope of evaluation in statistical modeling scenarios.

## Application to insulin resistance data

In the study by Malagón et al. (6), it is stated that insulin resistance (IR) is strongly associated with type 2 diabetes, cardiovascular disease, and stroke, among other conditions. The study obtains and validates various surrogate IR indices and, using a computational approach, predicts IR using the Matsuda index as a reference. It involves diagnostic accuracy assessments for different surrogate indices in young, non-diabetic adult men. One of the results, used below to illustrate the application of the Bayesian Youden index derived from Bayesian sensitivity and specificity, having demonstrated their equivalence to *PPV* and *NPV* respectively, pertains to the HOMA-IR index. HOMA-IR is generally more convenient in clinical practice than the Matsuda index because it only requires fasting glucose and insulin measurements, making it simpler, cheaper, and easier to apply routinely. In contrast, the Matsuda index requires a complete oral glucose tolerance test with multiple samples. Although HOMA-IR is used for illustration, Table 2 shows results for other indices from the same study to illustrate different scenarios, noting that in this study, the prevalence was P=0.484. Applying the algorithm proposed in the study yielded a sensitivity of $C_{se}=0.910$ and a specificity of $C_{sp}=0.980$, resulting in a Youden index value of $C_{Y}=0.890$.

**Table 2:** Properties of accuracy metrics relevant to their interpretation in practice.

Metric	Function of *P*	Built from *B_se/_B_sp_*	Statement about negative class
*C_Y_*	No (invariant)	No	Yes
*DOR*	No (invariant, proved)	No	Yes
*BA*	No (invariant)	No	Yes
*F*1	Yes (via *B_se_* only)	Partially(*B_se_* only)	No
*MCC*	Yes(correlation, no conditional probability)	No	Yes
*B_y_*	Yes	Yes	Yes

Since the prevalence of the condition for Table 1 is defined as $P=n(IR_{O})/n$, where both values are fixed (independent of the algorithm used for classification), it is clear that $C_{Y}$ is independent of prevalence. Given that $n(IR_{O})=nP$, $n(NIR_{O})=n(1-P)$, $n(TP)=C_{se}n(IR_{O})=nPC_{se}$ and $n(TN)=C_{sp}n(NIR_{O})=n(1-P)C_{sp}$, we have:

$$C_{Y}=\frac{n(TP)}{n(IR_{O})}-\frac{n(FP)}{n(NIR_{O})}=\frac{nPC_{se}}{nP}-\frac{n(1-P)-n(1-P)C_{sp}}{n(1-P)}=C_{se}+C_{sp}-1$$

Thus, $C_{Y}$ is independent of *P*, therefore, in the case of HOMA-IR, $C_{Y}=0.89$ will always have the same value. For the case of $B_{Y}$, but expressed as a function of prevalence, we have:

$$PPV=\frac{PC_{se}}{PC_{se}+(1-C_{sp})(1-P)}=B_{se}$$

$$NPV=\frac{(1-P)C_{sp}}{P(1-C_{se})+C_{sp}(1-P)}=B_{sp}$$

thus, $B_{Y}$ is written as:

$$B_{Y}=\frac{P(1-P)C_{Y}}{\left[PC_{se}+(1-C_{sp})(1-P)][P(1-C_{se})+C_{sp}(1-P)\right]}$$ (9)

which, through simplification in terms of $C_{Y},$ is equivalent to:

$$\;B_{Y}=\frac{P(1-P)C_{Y}}{\left[C_{se}-(1-P)C_{Y}][C_{sp}-PC_{Y}\right]}$$ (10)

which depends on prevalence. This is important when evaluating algorithm performance, as the observed dataset is not always balanced, which relates to a case where P≠0.5. From Equation 9, if P=0.5 it can be shown that by setting $C_{se}=C_{sp}$, we obtain $B_{Y}=C_{Y}$.

In the context of binary classification with imbalance, the index $C_{Y}$ has a fundamental limitation: by weighting both classes equally, it does not adequately penalize poor performance on the minority class. In contrast, the index $B_{Y}$implicitly incorporates the observed prevalence in the data, thereby reflecting the classifier's actual performance in the specific distribution of the problem. This characteristic is especially relevant when the prevalence in the evaluation dataset mirrors the expected distribution in production, as is the case in insulin resistance detection where the imbalance between resistant and non-resistant individuals is inherent to the clinical problem. Mathematically, $B_{Y}$ as expressed in Equation 10 highlights its nature as a weighted version of the classical Youden index $C_{Y}$adjusting for class distribution and marginal classification probabilities. Consequently, for problems with significant imbalance, $B_{Y}$ provides a more realistic evaluation of the classifier's clinical value than prevalence-independent metrics like $C_{Y}$ or accuracy. This dependence on prevalence in $B_{Y}$ does not constitute a limitation but rather a methodological advantage. The imbalance observed in our dataset (prevalence of resistance ≠ 50%) reflects the true distribution of the clinical phenomenon, and the algorithm will operate within this distribution in future applications. Therefore, $B_{Y}$automatically penalizes poor performance in the minority class: if the classifier generates many false positives (a common problem when the positive class is in the minority), the *PPV* decreases drastically, thereby reducing the composite index even if specificity remains high. In contrast, the classical Youden index $C_{Y}$ can maintain high values through high specificity even when detection of the minority class is poor, resulting in a potentially misleading evaluation of the classifier's true clinical value.

**Figure 2:**
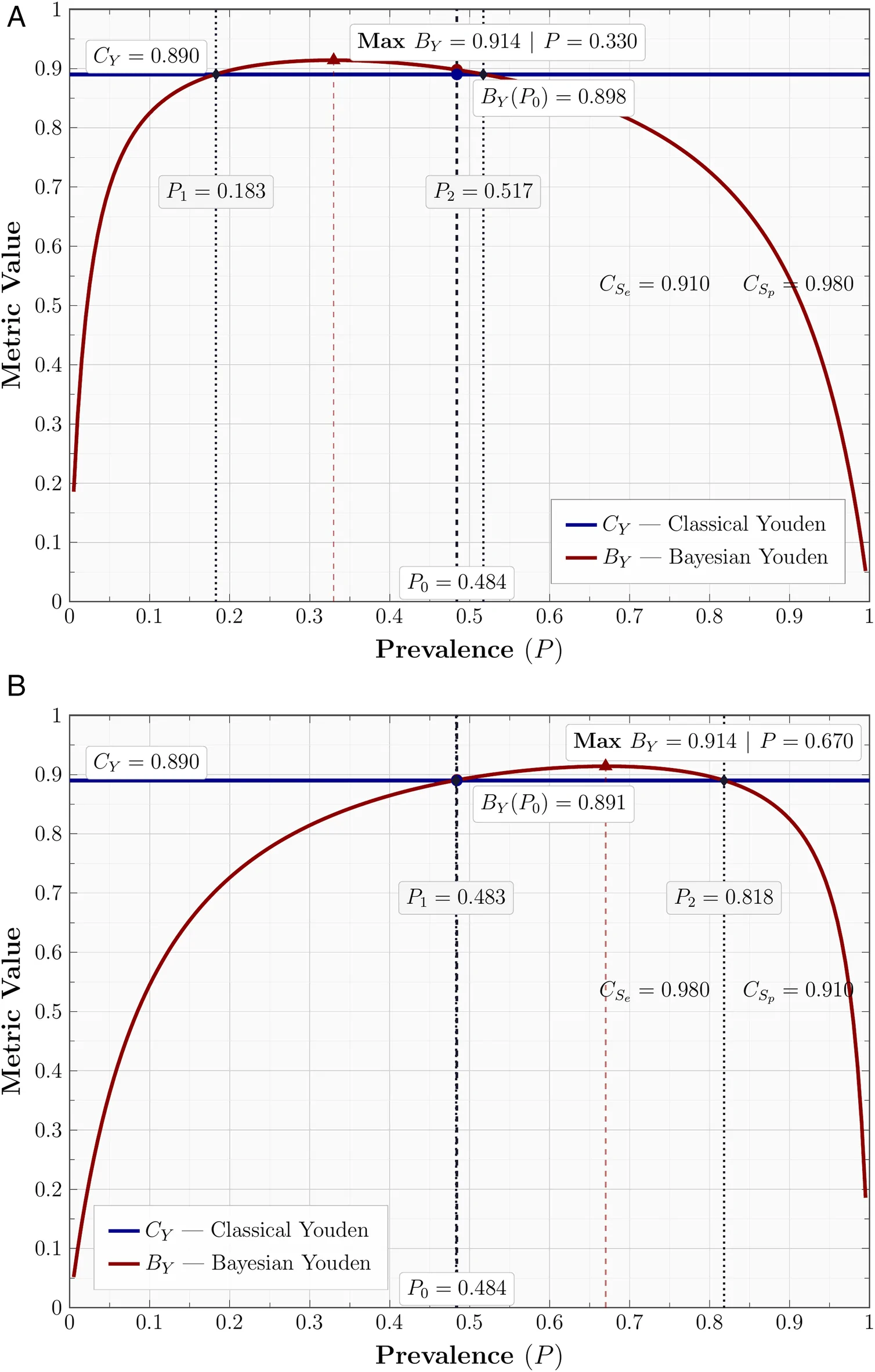
Analysis of $B_{Y}$ and $C_{Y}$ dependence on prevalence in binary classification performance. **(A)** (Original label) and **(B)** (swapped class labels).

It is also interesting to consider, as illustrated in Figure 2, a relevant asymmetric property of $B_{Y}$ when $C_{se}\neq C_{sp}$, where the function is not symmetric with respect to prevalence. In other words, $B_{Y}(P)\neq B_{Y}(1-P)$. This asymmetry, which can be demonstrated analytically, arises because the denominator in $B_{Y}$is symmetric only when $C_{se}=C_{sp}$. In our case, with $C_{se}=0.910$ and $C_{sp}=0.980$, the index $B_{Y}$ reaches higher values at low prevalences (*P* ≈ 0.25-0.40) than at their symmetric high-prevalence counterparts (*P* ≈ 0.60-0.75). This asymmetry is not an artifact but rather an informative characteristic, as it reveals that the classifier, being superior in specificity compared to sensitivity, will exhibit better operational performance in populations with low prevalence of resistance, where high specificity has a greater impact on predictive values. This information about the relationship between prevalence and expected performance is clinically relevant and is not captured by prevalence-independent indices like the classical Youden index. For these reasons, $B_{Y}$ is recommended as the primary evaluation metric, with the classical Youden index additionally reported to facilitate comparability with other studies.

Figure 2 illustrates the fundamental difference between the two indices. While the Youden index $C_{Y}$remains constant at 0.890 regardless of prevalence (blue horizontal line), reflecting the classifier's intrinsic capacity, the $B_{Y}\;$index varies substantially with prevalence (red curved line), reaching its maximum of approximately 0.914 at a prevalence of 33%. The proximity of $C_{Y}=0.890$ to the maximum $B_{Y}=0.914$ occurs precisely because the optimal prevalence for $B_{Y}$ (33%) is relatively close to 0.5, where many balanced datasets reside. Notably, at the observed HOMA-IR prevalence of 0.484, $B_{Y}=0.898$, demonstrating that even when data partitioning deviates from perfect balance, the Bayesian metric can maintain strong performance that approaches its theoretical maximum. Importantly, the $B_{Y}\;$demonstrates flexibility in imbalanced scenarios: at prevalences lower than the optimal 33% -potentially corresponding to minority class, partitions within the interval (L = 0.18; U = 0.52) yield $B_{Y}>C_{Y}$. If labels were reversed, the optimal prevalence would be 1-0.33 = 0.67 and the interval where $B_{Y}>C_{Y}$ would be (1-U;1-L) with the $max\;B_{Y}$ remaining the same in both cases (Figures 2A,B).

The Bayesian Youden index exhibits prevalence-dependent behavior that exceeds the classical threshold across moderate prevalence ranges, with the advantage being most pronounced near the optimal prevalence of 33%. This behavior reveals a fundamental constraint: no metric can compensate for severe class imbalance when base rates approach zero or unity, as BY drops below 0.20 at the distribution extremes (*P* < 15% or >80%), regardless of the sensitivity-specificity profile. The observed prevalence in the HOMA-IR example (*P* = 0.484) falls well within the range where the Bayesian formulation provides meaningful gains over the classical index, confirming that effective imbalance management requires joint optimization of evaluation metrics and classifier architecture rather than treating these factors independently. The evident asymmetry of the $B_{Y}$ curve with higher values near *P* = 25% than near *P* = 75%, analytically confirms that the classifier's superior specificity relative to sensitivity favors populations where accurate negative classification carries greater impact. This behavior highlights the complementary nature of the two indices: $C_{Y}$ as a prevalence-independent measure of inherent classifier ability, and $B_{Y}$ as a prevalence-dependent indicator of real-world utility that responds dynamically to different data partitioning scenarios.

The proposed index calculation for other alternative measures of insulin resistance from the article by Malagón et al. (6) appears at the following repository address (https://github.com/njgon/Bayesian-Youden-Index). In addition, the Shiny R script is provided that allows you to visualize the behavior of the classic Youden index as well as the Bayesian version.

## Intersection points between $B_{Y}$ and $C_{Y}$

Equating $C_{Y}=B_{Y}$ (Figure 2A) and operating algebraically, we obtain a polynomial in terms of $C_{se}$, $C_{sp}$ and *P*, and ordering for *P* ($P_{1}<P_{2}$) we can obtain the roots:

$$P_{1}=\frac{C_{sp}}{C_{se}+C_{sp}}$$ (11)

$$P_{2}=\frac{1-C_{sp}}{2-C_{se}-C_{sp}\;}$$ (12)

This can be verified in Figure 2 ($P_{1}\in$[0,1]; $P_{2}\in$[0,1]) with $C_{se}=0.91$, $C_{sp}=0.98(C_{Y}=B_{Y}=0.89)$. The prevalence rates in these cases correspond to $P_{1}=0.518$ and $P_{2}=0.182$. If any of the substitute insulin resistance indices are reached $C_{se}=1$ and $0<C_{sp}<1$, it turns out $P_{2}=1$ , and when $C_{sp}=1$ and $0<C_{se}<1,\;P_{2}=0$. When $C_{sp}=C_{se}=1$, then $P_{1}=P_{2}=0.5$. An interval (*I*) is generated only when $P_{1}\neq P_{2}$ with $I\in [min(P_{1},P_{2}),max(P_{1},P_{2})]$ which results in $B_{Y}\;\;C_{Y}$, with higher performance of the evaluated algorithm when $\max B_{Y}\in I$. In the case of the HOMA-IR index, I∈[0.182,0.518] with observed prevalence of $P_{o}=0.484$ and $C_{Y}=0.89$, therefore, a partition into classes that is unbalanced at the extremes of *I* yields a $C_{Y}=0.89$ while $B_{Y}$ reaches its maximum at $\max B_{Y}=0.914$ with optimal prevalence (partition) $P_{opt}=0.33.$ A researcher can not only control the labels of the classes defined to subject the algorithm and finally choose a partition that guarantees that $B_{Y}\;C_{Y}\;\;T\;,$ where *T* represents a desired threshold and that the partition associated with the prevalence in algorithmic performance can maximize a bivariate utility function with penalty parameter (18), or establish a convex constraint (19) using the desired minimum threshold by establishing a maximum deviation of imbalance or establishing a Pareto boundary (20).

An even more interesting result, which prevents the proposed measure from being regarded as a mere artificially generated metric seeking an analogy with the $C_{Y}$ index, is associated with the eigenvalue analysis of the resulting confusion matrix.

## Eigen-analysis of the confusion matrix and its relationship with $B_{Y}$

The purpose of this section is to test whether the coincidence between $B_{Y}$ and $C_{Y}$ at $P_{1}$ and $P_{2}$ (Equations 11, 12) is a property of the particular algebraic form chosen for $B_{Y}$, or whether it reflects a structural property of the confusion matrix itself, independent of that choice. The trace of Confusion Matrix M(P) (Equation 13) aggregates overall correctness (prevalence-weighted true positives and true negatives), and its determinant is directly proportional to $C_{Y}$ (Equation 14); examining the dominant eigenvalue of M(P) therefore offers a route to the same critical prevalence that does not presuppose the Bayesian derivation of $B_{Y}$ at all. That this independent route reproduces exactly $P_{1}$ and $P_{2}$ is the evidence that $B_{Y}$ is structurally, not merely notationally, related to $C_{Y}$.

An eigenvalue analysis of the binary confusion matrix is developed to identify underlying structures related to sensitivity and specificity associated with the algorithm's performance under variations in class distribution (21). Since the matrix summarizes the frequencies of *TP*, *FP*, *FN*, and *TN*, its eigenvalues reflect the magnitude of the dominant modes of error or correctness, which helps to assess the stability of the classifier in the face of changes in sample size and class imbalance. This provides a geometric and algebraic perspective that complements traditional metrics, with the ultimate purpose of linking the result to the proposed Bayesian Youden index.

Based on the Confusion Matrix (M(P)) as a function of *P,* $C_{se}$*,* $C_{sp}$ and sample size n:

$$M(P)=\left[\begin{array}{cc} n(TP) & n(FP)\\ n(FN) & n(TN) \end{array}\right]=n\left[\begin{array}{cc} PC_{se} & \left(1-P)(1-C_{sp}\right)\\ P(1-C_{se}) & (1-P)C_{sp} \end{array}\right]$$

The trace and determinant of M(P) are given by

$$tr(M(P))=n(PC_{se}+(1-P)C_{sp}))\;\;\;\;\;\;\;\;\;\;\;\;\;\;\;\;\;\;\;\;\;\;\;\;\;\;\;\;\;\;\;\;\;$$ (13)

$$det(M(P))=n^{2}P(1-P)C_{Y}\;\;\;\;\;\;\;\;\;\;\;\;\;\;\;\;\;\;\;\;\;\;\;\;\;\;\;\;\;\;\;\;\;\;\;\;\;\;\;\;\;\;\;\;\;\;\;$$ (14)

with det(M(P)≠0). The characteristic equation of M(P) is given by

$$\lambda_{M(P)}^{2}-tr(M(P))\lambda_{M(P)}+det(M(P))=0\;\;\;\;\;\;\;\;\;\;\;\;\;\;\;\;\;\;\;\;\;\;\;\;\;\;$$ (15)

whose extremum can be shown to be located at

$$\lambda_{M(P)}^{*}=\frac{n}{2}(PC_{se}+(1-P)C_{sp})=\frac{1}{2}tr(M(P))\;\;\;\;\;\;\;\;\;\;\;\;\;\;\;\;\;\;\;$$

which, when substituted into Equation 15 and saving the result in $\;\Phi (P,C_{se},C_{sp})$ we have $\Phi (P,C_{se},C_{sp})=det(M(P))-\frac{1}{2}tr^{2}(M(P))$, where $\Phi (P,C_{se},C_{sp})$ is function on $P,\;C_{se}$ and $C_{sp}.\;\mathrm{By}\;\mathrm{ordering}\;\mathrm{with}\;\mathrm{respect}\;\mathrm{to}\;P$, we can express:

$$\Phi (P,C_{se},C_{sp})=P^{2}f(C_{se},C_{sp})+Pg(C_{se},C_{sp})+h(C_{se},C_{sp})$$

with $f(C_{se},C_{sp})=(C_{se}-C_{sp}{)}^{2}-4(C_{se}+C_{sp}+1)$; $g(C_{se},C_{sp})=\;$ $2C_{sp}(C_{se}-C_{sp})-4(C_{se}+C_{sp}-1)\;$ and $h(C_{se},C_{sp})=C_{sp}^{2}$.

By making the structural separation as

$$\Phi (P,C_{se},C_{sp})=Q_{P}(C_{se},C_{sp})+L_{P}(C_{se},C_{sp})+c(P)$$

where the homogeneous quadratic part is as

$$Q_{P}(C_{se},C_{sp})=P^{2}(C_{se}-C_{sp}{)}^{2}+P(2C_{se}C_{sp}-2C_{sp}^{2})+C_{sp}^{2}$$

and the linear part as

$$L_{P}(C_{se},C_{sp})=-4(P^{2}+P)(C_{se}+C_{sp})$$

and the constant as

$$c(P)=-4P^{2}+4P.$$

Because Φ depends on $C_{se}$ and $C_{sp}$ only through their sum and their ratio, as shown above, a natural reparametrization is to separate a scale coordinate $s=C_{se}+C_{sp}$ from a direction coordinate $t=C_{sp}/(C_{se}+C_{sp})$, precisely a barycentric coordinate on the segment joining $C_{se}$ and $C_{sp}$ (33). This substitution reduces a two-parameter optimization to a one-parameter optimization in *t*, which is what makes the closed-form critical prevalence in Equation 11, 12 obtainable directly. It is clear that the linear part depends on $C_{se}+C_{sp}$, thus, using natural barycentric coordinates (22) (This expression depends only on the sum $C_{se}+C_{sp}$ and not on individual values) with s≠0, we have $C_{se}=s(1-t)$ and $C_{sp}=st$, which are the coordinates of the segment [$C_{se},C_{sp}$], in this way, it follows that

$$Q_{P}(C_{se},C_{sp})=Q_{P}(s,t)=2t-4t^{2}$$

$$L_{P}(C_{se},C_{sp})=L_{P}(s,t)=-4(P^{2}+P)s$$

thus, the total form of $\Phi (P,C_{se},C_{sp})$ is now written as

$$\Phi (P;s,t)=s^{2}F(P,t)-4(P^{2}+P)s+c(P)$$

where $F(P,t)=P^{2}(1-2t{)}^{2}+P(2t-4t^{2})+t^{2}.$ Thus, *s* is a scale parameter and *t* is the barycentric direction (recall that structural extrema cannot depend on scale, so the critical parameter *P* must arise from the dependence on t). Now, taking the gradient with respect to (s,t) we obtain that

$$\begin{array}{c} \frac{\partial \Phi (P;s,t)}{\partial s}=2sF(P,t)-4(P^{2}+P)=0\\ \frac{\partial \Phi (P;s,t)}{\partial t}=s^{2}\frac{\partial F}{\partial t}=0\;\;\;\;\;\;\;\;\;\;\;\;\;\;\;\;\;\;\;\;\;\;\;\;\;\;\;\;\;\;\;\;\; \end{array}$$

and since s≠0, the actual condition is $\frac{\partial F}{\partial t}=0$. The explicit barycentric derivatives are:

$\frac{\partial F(P,t)}{\partial t}=t(8P^{2}-8P+2)+(-4P^{2}+2P)$ and, by making $t(8P^{2}-8P+2)=4P^{2}-2P$ we obtain $t=\frac{P}{2P-1}$. Inverting the problem in a natural way, and recalling that *P* is the parameter while *t*, is the geometric datum, we invert the relation with P=t, in this way

$$P_{1}=t=\frac{C_{sp}}{C_{se}+C_{sp}}$$

and under the complementary affine transformation of the confusion matrix, it follows that

$$P_{2}=\frac{1-C_{sp}}{2-C_{se}-C_{sp}}$$

which coincides with the values ($P_{1},P_{2}$) obtained in Equations 11, 12.

The variables initially considered, namely $\left(C_{se},C_{sp}\right)$, do not belong to the space $R^{2}$, but rather to a compositional space. Recall that the confusion matrix with fixed *n* and row values was designed by the study. In the polynomial $\Phi (P,C_{se},C_{sp})$, if one does

$$\left(C_{se},C_{sp})\mapsto (rC_{se},rC_{sp}\right)$$

Thus, the quadratic terms scale as $r^{2}$, the linear terms scale as *r* the linear terms as *r*, and the critical parameter *P* does not change. This invariance is precisely the organizing principle of compositional data analysis (23): once a quantity depends on $(C_{se},C_{sp})\;$ only through a common rescaling-invariant combination, it is the relative proportion between them, not their absolute magnitude that carries information about the location of the structural extremum.

What this invariance implies is that the relevant information lies in the direction of the vector $\left(C_{se},C_{sp}\right)$ rather than in its magnitude, and therefore every direction in $R_{+}^{2}$ is parameterized by a ratio of the form $C_{se}/(C_{sp}+C_{se})$, rather than by $C_{se}$ or $C_{sp}$ alone. Hence, for the specific case of sensitivity and specificity, we can say that

$$\left(C_{se},C_{sp})\in R_{+}^{2}\Rightarrow (C_{se},C_{sp})=s(1-t,t\right)$$

Thus, *s* is an artificial scale parameter and *t* represents the real information, which is mathematically legitimate because we would be going from $R_{+}^{2}\to P_{+}^{1}$ (from the positive plane to the positive projective space), and in this space, the natural coordinates are quotients.

Although the maximum eigenvalue $\lambda_{max}^{*}$ has no direct interpretation, it can be interpreted structurally: thus, $\lambda_{max}^{*}\;$ r represents a global measure of the dominant structural magnitude of the classification. The value $\lambda_{max}^{*}$ depends on the sum of TN and TP as well as on the cross-error product FN and FP. When a perfect classifier is achieved, that is, FN=FP=0, $\lambda_{max}^{*}=\max(TN,TP)$, which makes the system completely aligned with the truth, and when FN and FP are large, $\lambda_{max}^{*}$ does not distinguish the classifier's performance, thus, $\lambda_{max}^{*}\;$ is a measure of structural dominance between correct predictions and cross-errors. Since the data comes from a compositional structure by study design, $\lambda_{max}^{*}$ could be seen as the “principal mode” of the classification structure. Since the data comes from a compositional structure by study design, $\lambda_{max}^{*}$ could be seen as the “principal mode” of the classification structure. From the matrix M(P), when $C_{se}=C_{sp}=1$, $\lambda_{max}^{*}\;=\;max(P,1-P)$. From the matrix M(P), when $C_{se}=C_{sp}=1$, $\lambda_{max}^{*}=max(P,1-P)$. From the result of Equation 14, it can be noted how $\lambda_{max}^{*}$ is a spectral function of $C_{Y}$. Thus, when the characteristic polynomial is maximized, the critical point occurs when the matrix is spectrally balanced (without loss of generality), that is, $(1-P)C_{sp}=PC_{se}$, which means that the spectral mass of TN and TP is balanced, which means that the prevalence-weighted mass of true negatives and true positives is balanced. At this point, the population-conditioned index ($C_{Y}$) and the prediction-conditioned index ($B_{Y}$) are quantifying the same discriminative content in the confusion matrix, precisely the condition under which Equations 11, 12 showed the two indices to coincide algebraically.

## Geometric interpretation in the 2 × 2 Simplex

In the compositional structure of the 2 × 2 confusion matrix, $C_{Y}$ measures vertical separation (compares within each true state—separation between rows), $B_{Y}$ measures horizontal separation (compares within the algorithmic classifier—separation between columns), and $\lambda_{max}^{*}$ measures the dominant direction. Thus, when $(1-P)C_{sp}=PC_{se}$, the matrix lies on the structural bisector of the compositional space, so the result found in the coincidence between Equations 11, 12 with the eigen-analysis results for with the eigen-analysis results for $\lambda_{max}^{*}$, produces the prevalence point where population discrimination aligns with predictive discrimination (the dominant spectral direction is maximized), thus obtaining a point of algebraic symmetry of the system. This coincidence is more than decorative or casual from trying to make an analogy with $C_{Y}$ from predictive values, but rather it is an attempt to achieve population and predictive discrimination simultaneously.

The index $B_{Y}$ reflects the intrinsic quality of the classifier and the predictive values reflect the contextual utility (how reliable the predictions are in a specific population). A good classifier should show consistency in both indicators: high $C_{Y}$ and adequate predictive values. If there is a discrepancy, it means that the model is limited by class imbalance or by prevalence. However, at $P_{1}$ or $P_{2}$ the asymmetry induced by prevalence associated with imbalanced classes is nullified, and although in clinical practice it is not widely used due to the observed prevalence (the reality that prevails), from a theoretical point of view it is very relevant, since the coincidence identifies the prevalence where the test is mathematically balanced and its internal coherence is maximized.

Since the early development of machine learning, balanced classes have been recommended as a convenient condition for metrics such as accuracy, error rate, and symmetric losses (24). However, the results presented here suggest that this symmetry should not be treated as a universal requirement. While $C_{Y}$ measures separation conditional on the true class label, $B_{Y}$ measures separation conditional on the prediction, better captures the real utility of the classifier by reflecting the operational reality of the deployment context, revealing that there exists a more fundamental symmetry point than simple 50/50 balance, namely, the prevalence at which the Bayesian and classical formulations coincide. From this perspective, artificially rebalancing classes is not inherently wrong, but it is unnecessary when $B_{Y}$ already demonstrates adequate posterior performance under the observed prevalence. Intervening at the data level in such cases risks altering the genuine geometry of the classifier by discarding an intrinsic equilibrium determined by the sensitivity-specificity profile, replacing it with an artificial distribution that may not reflect the population the classifier will actually encounter. The proposed framework therefore suggests that the decision to rebalance should follow, rather than precede, a prevalence-aware evaluation of classifier performance, with $B_{Y}$ providing precisely the diagnostic needed to make that determination in a principled way.

## Analytical comparison with alternative imbalance-aware metrics

The claim that $B_{Y}$ offers a genuine structural advantage over other imbalance-aware summaries of the confusion matrix, rather than an advantage that could equally be captured by existing measures can be established analytically, without recourse to simulation, by examining how each measure is constructed from $C_{se}$, $C_{sp}$, and *P*.

A measure is here termed “predictively grounded” if, like $B_{se}$ and $B_{sp}$, it is conditioned on the observed classification and expresses a probability of the unobserved true state. This conditioning is the one relevant to decisions made at the moment a result is obtained. $B_{Y}$ is predictively grounded by construction, since it is built entirely from $B_{se}=PPV$ and $B_{sp}=NPV$.

- Diagnostic odds ratio. Using $TP=nPC_{se}$, $TN=n(1-P)C_{sp}$, $FP=n(1-P)(1-C_{sp})$, $FN=nP(1-C_{se})$, the diagnostic odds ratio reduces to
  $$DOR=\frac{C_{se}C_{sp}}{\left(1-C_{se})(1-C_{sp}\right)}$$

where every factor of *P* and (1−P) cancels between numerator and denominator. DOR is therefore, provably, a function of $C_{se}$ and $C_{sp}$ alone: like $C_{Y}$, it is invariant to prevalence and cannot register the deployment-dependent behavior that motivates $B_{Y}$. Unlike $C_{Y}$, however, DOR is not bounded and has no natural relationship to a Youden-type [−1, 1] scale.

- Balanced accuracy (BA)*.* By definition, $BA=(C_{se}+C_{sp})/2=C_{Y}/2+1/2$, an affine function of $C_{Y}$ alone. It is therefore prevalence-invariant for exactly the same algebraic reason as $C_{Y}$, and inherits none of the predictive grounding of $B_{se}$ and $B_{sp}$.
- F1 *score*. Writing F1 in terms of precision and recall,
  $$F1=\frac{2B_{se}C_{se}}{B_{se}+C_{se}}$$

since precision is by definition $B_{se}(PPV)$ and recall is $C_{se}$. F1 therefore, incorporates exactly one predictively grounded quantity $\left(B_{se}\right)$ alongside one that is not $\left(C_{se}\right)$, and — because its defining expression 2TP/(2TP+FP+FN) contains no TN term at all, it carries no information about $B_{sp}(NPV)$ whatsoever. F1 is consequently silent, by construction, on the predictive reliability of a negative classification, which is inappropriate whenever both classes carry clinical weight, as in insulin resistance screening.

- *Matthews correlation coefficient* (MCC). Substituting the same expressions for TP, TN, FP, FN in
  $$MCC=\frac{TPTN-FPFN}{\sqrt{\left(TP+FP)(TP+FN)(TN+FP)(TN+FN\right)}}$$

the numerator simplifies to $TPTN-FPFN=n^{2}P(1-P)C_{Y}$, precisely $n^{2}$ times the determinant identity already established in Equation 14, while the denominator factors as $n^{2}\sqrt{P(1-P)D_{1}D_{2}}$, with $D_{1}=PC_{se}+(1-P)(1-C_{sp})$ and $D_{2}=(1-P)C_{sp}+P(1-C_{se})$, the marginal probabilities of a positive and a negative classification, respectively. This yields the closed form:

$$MCC=C_{Y}\sqrt{\frac{P(1-P)}{D_{1}D_{2}}}$$

MCC is therefore genuinely prevalence-sensitive, unlike DOR and balanced accuracy, but it remains, by this derivation, the classical Youden index rescaled by a symmetric function of the true-class and predicted-class marginals, i.e., the phi coefficient of the 2 × 2 table (reference). It quantifies the correlation between predicted and true labels, not a conditional probability of the true state given a classification, and it does not decompose into $B_{se}$ and $B_{sp}$ in the way $B_{Y}$ does; consequently, MCC cannot be read directly as a predictive confidence statement at deployment.

Table 2 summarizes these results. Among the five measures compared, $B_{Y}$ is the only one that is simultaneously (i) sensitive to prevalence, and (ii) constructed entirely from probabilities conditioned on the observed classification. This distinction is not a matter of degree that a numerical comparison could soften: DOR and BA are prevalence-invariant as a matter of algebraic identity, F1 omits the negative-class predictive statement as a matter of its defining formula, and MCC, while prevalence-sensitive, is an association measure rather than a conditional probability. The advantage of $B_{Y}$ over each of these alternatives is therefore structural rather than empirical.

Taken together, these results show that the advantage of the proposed index is not one of degree, to be confirmed or weakened by empirical comparison across simulated scenarios, but one of construction. Among the compared measures, only the proposed index is simultaneously sensitive to prevalence and built entirely from probabilities conditioned on the observed classification, the direction of conditioning relevant to a decision made at the moment a result is obtained. The *DOR* and *BA* are provably prevalence-invariant; the *F*1 score is provably silent on the predictive reliability of a negative classification; and the MCC, while prevalence-sensitive, remains an association measure rather than a conditional probability. None of these limitations depends on the particular dataset or operating point chosen, and none would be resolved by evaluating the measures on additional simulated data, since each follows directly from the algebraic definition of the measure itself (25, 26). At the same time, it is worth noting that the practical interpretation of the MCC can be affected by factors such as class imbalance and the quality of the reference standard used for validation. As Foody (27) points out, when the reference standard is imperfect, the apparent MCC may diverge substantially from its true value, with the direction and magnitude of the bias depending on whether errors are independent or correlated with those of the classifier. In particular, a relatively high MCC may be observed even for a poor classifier under certain conditions, which suggests that apparent MCC values should be interpreted with care, especially when the gold-standard assumption is not met. These considerations do not undermine the theoretical properties of the MCC, but they do highlight that, like any summary metric, it benefits from contextual interpretation and, where possible, correction for reference data imperfections. The proposed index, by contrast, offers a direct conditional-probability reading that aligns with the decision-making setting, without replacing the need for careful validation practices.

## Important aspects of algorithmic deployment

The application of Bayes' theorem to estimate predictive values across different populations rests on a fundamental assumption: that an algorithm with fixed parameters maintains constant sensitivity and specificity regardless of the population context in which it is applied, with only the predictive values varying as a function of local prevalence. This assumption implies that the intrinsic metrics of the model, sensitivity and specificity, are stable properties of the algorithm itself, analogous to the characteristics of a physical diagnostic test (12). To support the practical implementation of this framework, an interactive tool is available that allows users to explore the full $B_{Y}$ framework directly from a reported confusion matrix, without requiring additional software or programming expertise. The application includes five components: a graphical display of $C_{Y}$ and $B_{Y}$ as functions of prevalence with automatic identification of the maximum $B_{Y}$ and the intersection points $P_{1}$ and $P_{2}$; a parametric bootstrap module for uncertainty quantification that produces 95% confidence intervals for all key statistics from user-supplied confusion matrix counts; a reconstructed confusion matrix with all derived classical and Bayesian metrics at the observed prevalence; a numerical table of metric values across the full prevalence range; and a reference panel describing the theoretical foundations of the indices. The tool is publicly accessible at https://nair-gonzalez.shinyapps.io/Bayesian_Youden_App/).

## Results for other surrogate indices of insulin resistance

The observed values of $C_{Y}\;$ and $B_{Y}$ for the different insulin resistance indices, whether as direct measures, indirect measures, or those derived through mathematical operations, as well as other markers used, show a remarkable similarity. This is due to the observed prevalence of P=0.484 being very close to the equilibrium point of P=0.5. This proximity to class balance explains why most indices maintain practically identical values across both metrics. However, the true utility of distinguishing between metrics becomes evident in clinical contexts or algorithmic performance evaluations where prevalences are imbalanced, but where $B_{Y}\geq C_{Y}$. In populations with prevalences in this region (when $P_{1}$ and $P_{2}\;$ are generated, since when $C_{sp}=C_{se}=1$ only $P_{1}$ is generated, just as when only one of the metrics, sensitivity or specificity, reaches unity) both metrics can differ substantially, reflecting how the clinical performance of the classifier (measured by predictive values) diverges from its intrinsic discriminative capacity (measured by sensitivity and specificity). This divergence is particularly relevant in general population screening studies (low prevalence) or in high-risk patient cohorts (high prevalence), where selecting the optimal cutoff point must consider not only discriminative capacity but also predictive utility.

As you can see in Tables 3–5, $P_{2}$ is not defined since, from Equation 12, the denominator $2-C_{se}-C_{sp}=0$, as well as when $P_{2}=0$ when $C_{sp}=1.$ The results obtained for the selection of insulin resistance indices allowed again the choice of the direct index METS-IR, the indirect indices TyG-WC and TyG-BMI, BRI as a related indirect measure, and Leptin as an additional marker (20). All measures in the following tables have been rounded up to three significant digits per space limit.

**Table 3:** Direct Measures of Insulin Resistance (IR).

Index	*P*_1_	*P*_2_	*C*_Y_	*B*_Y_
HOMA-IR (Homeostatic Model Assessment of IR)	0.52	0.18	0.89	0.90
QUICKI (Quantitative Insulin Sensitivity Check Index)	0.49	0.67	0.94	0.94
METSIR (Metabolic Score for IR)	0.50	-	1.00	1.00
Mcai (Metabolic Analysis Index)	0.49	0.60	0.89	0.89

**Table 4:** Indirect Indices (without insulin measurement).

Index	*P*_1_	*P*_2_	*C*_Y_	*B*_Y_
TyG-Wc (Triglyceride-Glucose•Index-Waist circumference)	0.50	-	1.00	1.00
TyG- BMI (TyG×Body Mass Index)	0.50	-	1.00	1.00
TyG_WHtR (TyG×Waist-to-height ratio)	0.51	0.00	0.98	0.98
AIP (Atherogenic Index Of Plasma)	0.52	0.38	0.74	0.74

**Table 5:** Indirectly Related Indices.

Index	*P*_1_	*P*_2_	*C*_Y_	*B*_Y_
CMI (Cardiometabolic Index)	0.54	0.00	0.84	0.87
CI (Conicity Index)	0.49	0.59	0.83	0.83
ABSI (A Body Shape Index)	0.53	0.46	0.05	0.05
Bri (Body Roundness Index)	0.51	0.00	0.98	0.98

## Uncertainty quantification via parametric bootstrap (HOMA-IR)

Although $B_{Y}$, its maximum, and the intersection points $P_{1}$ and $P_{2}$ admit closed-form expressions derived directly from the confusion matrix, they are functions of the estimated sensitivity and specificity, which are themselves proportions subject to sampling variability. To characterize the precision of these point estimates we employed a parametric bootstrap procedure grounded in the binomial sampling model that governs the confusion matrix.

### Probabilistic model

Let $n(IR_{O})=n(TP)+n(FN)$ denote the total number of truly positive cases and $n(IR_{A})=n(TP)+n(FP)$ the total number of truly negative cases. The observed counts of correct classifications follow independent binomial distributions:

$$TP∼Binomial(n(IR_{O}),C_{se})\;\text{and}\;TN∼Binomial(n(IR_{A}),C_{sp})$$

where $C_{se}$ and $C_{sp}$ are the true but unknown classical sensitivity and specificity. This independent-binomial structure follows from the sampling design: conditional on the true-class totals $n(IR_{O})$ and $n(NIR_{O})$, fixed by how the reference classification was ascertained, the number of correct classifications within each true-class group is a sum of independent Bernoulli trials with success probability $C_{se}$ or $C_{sp}$ respectively. This conditional-independence structure is precisely what sensitivity and specificity are defined to measure, which is why the two margins were modeled as independent binomials rather than the four confusion-matrix cells as a single multinomial, a multinomial model would not privilege the true-class conditioning on which $C_{se}$ and $C_{sp}$ are defined. Point estimates for **s**ensitivity and specificity are the maximum likelihood estimators under this model (11). To incorporate uncertainty in the $P_{1}$ and $P_{2}$ points for the case of the HOMA-IR index, the parametric bootstrap procedure was used. For each of B = 2,000 replications, bootstrap counts were generated as:

$$TP^{*}∼Binomial(n(IR_{O}),\;C_{se}^{*})\;\text{and}\;TN^{*}∼Binomial(n(IR_{A}),C_{sp}^{*})$$

yielding bootstrap estimates $\;C_{se}^{*}$ and $C_{sp}^{*}$. From each pair the following statistics were recomputed analytically: the classical Youden index $C_{Y}^{*}\;=\;C_{se}^{*}+C_{sp}^{*}-1$; the Bayesian Youden index $B_{Y}^{*}$ evaluated over a grid of 999 prevalence values in (0,1); the maximum of $B_{Y}^{*}$ and the prevalence at which it is attained; and the intersection points $P_{1}^{*}$ and $P_{2}^{*}$ defined as the prevalences where $B_{Y}^{*}\;=\;C_{Y}^{*},$ located as the midpoints of sign-change intervals in the difference $B_{Y}^{*}-C_{Y}^{*}$ with a tolerance of 0.0015. Replications yielding degenerate estimates $C_{se}^{*}\;=\;0$ or 1 and $C_{sp}^{*}\;=\;0$ or 1 were excluded from the summaries for the affected statistics. The random seed was fixed at 42 for reproducibility. Percentile-method 95% confidence intervals were constructed from the resulting bootstrap distributions. The observed confusion matrix for the HOMA-IR classifier corresponds to n=93 subjects, with n(TP)=41, n(FP)=1, n(FN)=4 and n(TN)=47. Table 6 reports the observed values of each statistic and their bootstrap 95% confidence intervals (B = 2,000 replicates bootstrap).

**Table 6:** Bootstrap 95% confidence intervals for different statistics (only the notation * is maintained in the results of the bootstrap resampling).

Statistic	Observed	CI-Lower	CI-Upper	Bootstrap SE	Valid replicates
$C_{Y}^{*}$	0.890	0.782	0.978	0.048	2,000
$Max\;B_{Y}^{*}$	0.913	0.823^a^	0.997^a^	0.056	2,000
P at $Max\;B_{Y}^{*}$	0.334	0.027	0.575	0.193	2,000
$P_{1}^{*}$	0.190	0.118	0.542	0.150	2,000
$P_{2}^{*}$	0.518	0.504	0.652	0.061	1,082

a BCa interval: [0.700, 0.995]; reproducible via the Robustness Check tab of the accompanying Shiny application.

The algorithmic value of $B_{Y}$ over $C_{Y}$ becomes apparent when the operating prevalence departs from balance. For the HOMA-IR classifier ($C_{se}=0.910$, $C_{sp}=0.980$, $C_{Y}=0.890),$ three prevalence regimes can be distinguished. At prevalences below $P_{1}=0.190$, $B_{Y}<C_{Y}$: the classical index overestimates classifier utility because a high specificity generates few false positives even by chance when the condition is rare, inflating the apparent performance. Between $P_{1}$ and $P_{2}=0.518$, $B_{Y}>C_{Y}$, with a maximum of $B_{Y}=0.913$ at P=0.334 [95% CI: (0.823, 0.997)]: in this intermediate regime the classifier genuinely outperforms what the prevalence-blind $\;C_{Y}$ suggests, because the moderate prevalence optimally leverages the asymmetry between sensitivity and specificity. Above $P_{2}$, $B_{Y}$ falls again below $C_{Y}$ as the growing share of positive cases erodes the negative predictive value. These transitions invisible to $C_{Y}$, directly inform deployment decisions: a screening program operating at a population prevalence of insulin resistance of 33% would correctly identify this classifier as performing above its classical benchmark, while one deployed in a low-prevalence context (e.g., P<0.19) would recognize the same classifier as operating below that benchmark despite identical sensitivity and specificity. The bootstrap confidence intervals for $P_{1}$ [(0.118, 0.542)] and $P_{2}$ [(0.504, 0.652)] quantify the uncertainty around these decision boundaries, providing actionable precision estimates that point estimates alone cannot convey.

To assess whether the percentile method is appropriate for this dataset, the empirical bootstrap distributions of $C_{Y}^{*}$, of $B_{Y}^{*}$ at the observed prevalence, and of $C_{Y}^{*}$ at its maximum were examined using the Robustness Check module of the accompanying Shiny application (see note to Table 6). All three distributions show moderate negative skewness (−0.42, −0.40, and −0.34, respectively), reflecting the small number of misclassifications: one false positive and four false negatives in a sample of 93 subjects. For $C_{Y}^{*}$, and for $B_{Y}^{*}$ at the observed prevalence, percentile and bias-corrected and accelerated (BCa) intervals were similar ($C_{Y}^{*}$: [0.782, 0.978] vs. [0.763, 0.957]; $B_{Y}^{*}$ at P=0.484: [0.806, 0.980] vs. [0.785, 0.961]), differing mainly by a small downward shift of both bounds consistent with the observed skew. For $B_{Y}^{*}$ at its maximum, however, the BCa interval was markedly wider on the lower side than the percentile interval reported in Table 6 [[0.700, 0.995] vs. [0.823, 0.997]].

This divergence corroborates, from an independent diagnostic, the geometric flatness of the $B_{Y}$ curve near its peak already noted above: the maximum is precisely the statistic for which the choice of interval method matters most, and we therefore report the BCa interval as the more conservative and appropriate summary for this quantity. As a further check on the sampling model itself, we compared this parametric (independent-binomial, fixed-margin) bootstrap against a non-parametric case-resampling bootstrap, resampling the n=93 subject-level records with replacement and recomputing the confusion matrix at each of B = 2,000 replications; percentile intervals from the two procedures agreed closely for all three statistics [e.g., $C_{Y}^{*}$: [0.782, 0.978] parametric vs. [0.794, 0.976] non-parametric], indicating that the independent-binomial sampling model does not materially distort the reported uncertainty relative to a multinomial-consistent resampling of the confusion matrix (28).

## Risk prediction of diabetes progression and domain generalization

To illustrate that Bayesian Youden is not confined to the insulin resistance domain, we applied it to published confusion matrices from an independent diabetes progression study using physical examination data from a Chinese hospital cohort (29). That study reports logistic regression classifiers for two sequential binary tasks, normal vs. prediabetes and prediabetes vs. diabetes, with markedly different class prevalences (P=0.65 and P=0.17, respectively), providing a natural test case for prevalence sensitivity. Applying our proposal, to the reported testing-set confusion matrices yielded $B_{Y}=0.46$ for the normal-to-prediabetes task and $B_{Y}=0.65$ for the prediabetes-to-diabetes task, compared to classical Youden values of $C_{Y}=0.34$ and $C_{Y}=0.28$. Three observations are noteworthy. First, $C_{Y}$ ranks the two classifiers as nearly equivalent and even inverts their ordering relative to $B_{Y}$, suggesting marginally better performance in the normal-to-prediabetes task; $B_{Y}$ correctly identifies the prediabetes-to-diabetes classifier as substantially superior once the asymmetric prevalence structure is taken into account. Second, the magnitude of the correction from $C_{Y}$ to $B_{Y}$, is proportional to the degree of imbalance: the low-prevalence task (P=0.17) undergoes a correction of +0.36, nearly three times the correction observed in the near-balanced task (P=0.65, correction +0.12), which is consistent with the theoretical behavior of the index. Third, this demonstration involves a domain, a dataset, a prevalence regime, and a clinical question entirely distinct from insulin resistance assessment, supporting the claim that $B_{Y}$ constitutes a general-purpose evaluation framework rather than a domain-specific adjustment. The full numerical decomposition, including Bayesian sensitivity, Bayesian specificity, and their components is presented in Table 7.

**Table 7:** Diabetes progression prediction domain using Logistic regression Model.

Statistic	Normal → prediabetes	Prediabetes → Diabetes
Prevalence P	0.6492 (majority class)	0.1739 (minority class)
$C_{se}$	0.9067	0.3027
$C_{sp}$	0.4295	0.9818
$C_{Y}$	0.3362	0.2845
$B_{se}$	0.7463	0.7778
$B_{sp}$	0.7131	0.8699
$B_{Y}$	0.4594	0.6477
Difference:	+0.1232	+0.3632

## Contextualisation and scope

The Bayesian Youden Index occupies a specific and well-defined position within the landscape of binary classification evaluation metrics and understanding that position is essential to interpreting its contribution correctly. The metrics most employed in imbalance-aware evaluation, Matthews Correlation Coefficient (MCC), balanced accuracy, *F*1-score, G-mean, and AUROC, are aggregate performance summaries (30). They collapse classifier behavior across all possible thresholds, or at a single fixed threshold, into a scalar that does not explicitly model the role of prevalence. $B_{Y}$, by contrast, is a threshold-level calibration framework: it expresses, as a continuous function of prevalence, the degree to which a classifier's posterior discriminative capacity, measured jointly through *PPV* and *NPV* exceeds chance. Its inputs are the four cells of a single confusion matrix and the class prevalence; its output is not a ranking of classifiers but a prevalence-dependent profile that identifies the operating conditions under which a given classifier's Bayesian performance surpasses, equals, or falls short of its classical benchmark. To make this distinction concrete: AUROC summarizes performance across thresholds but is insensitive to class imbalance and does not incorporate prevalence. MCC and *F*1-score are threshold-specific and imbalance-sensitive, but yield single numbers that provide no information about how performance evolves as the class distribution shifts. The diagnostic odds ratio (*DOR*) (31) can be shown algebraically to depend on sensitivity and specificity alone, with every factor of prevalence canceling between numerator and denominator; it is therefore, like the classical Youden index, invariant to prevalence, and it does not offer a bounded, Youden-like scale in exchange. Balanced accuracy averages sensitivity and specificity, which is algebraically equivalent to $C_{Y}$/2 + 0.5 and therefore inherits exactly the prevalence-blindness that $B_{Y}$ is designed to correct. None of these metrics answers the question $B_{Y}$ addresses: at what prevalence does the classifier's posterior performance peak, and across what range of class distributions does its Bayesian utility exceed its classical utility? $B_{Y}$ is therefore best understood as complementary to rather than competitive with aggregate metrics, practitioners would use AUROC or MCC to select among classifiers, and $B_{Y}$ to characterize the prevalence sensitivity of the selected classifier before deployment.

Regarding class imbalance specifically, the intersection points $P_{1}$ and $P_{2}$, the prevalences at which $C_{Y}$ and $B_{Y}$ coincide, serve as actionable decision boundaries rather than mere analytical curiosities. For the HOMA-IR classifier, $P_{1}=0.190$ and $P_{2}=0.518$ define three distinct operating regimes. Below $P_{1}$, the classical index overestimates classifier utility because high specificity generates few false positives even by chance when the condition is rare. Between $P_{1}$ and $P_{2}$, $B_{Y}$ exceeds $C_{Y}$, with a maximum of 0.913 at P=0.334, a moderately imbalanced setting where the asymmetry between sensitivity and specificity is optimally leveraged by the Bayesian formulation. Above $P_{2}$, $B_{Y}$ falls below $C_{Y}$ as the growing share of positive cases erodes the negative predictive value. These transitions, invisible to $C_{Y}$, directly inform deployment decisions: a screening program operating at a population prevalence of insulin resistance near 33% would correctly identify this classifier as performing above its classical benchmark, while one deployed in a low-prevalence context below $P_{1}$ would recognize the same classifier as operating below that benchmark despite identical sensitivity and specificity values. This behavior also clarifies when data-level balancing techniques such as SMOTE are and are not warranted (32): $B_{Y}$ provides a principled diagnostic that, when it reveals adequate posterior performance under the operational prevalence, makes artificial augmentation unnecessary and potentially counterproductive given its well-documented limitations including the generation of noisy synthetic samples in overlapping regions and the risk of overfitting to interpolated feature space.

Although the present study develops and validates $B_{Y}$ within the insulin resistance domain, the index is not domain-specific. Its sole inputs are the four cells of any confusion matrix and the class prevalence, quantities universally available across binary classification problems regardless of clinical context, dataset origin, or degree of imbalance. This means that any investigator reporting a confusion matrix can compute $B_{Y}$ immediately, without additional data collection, model retraining, or algorithmic intervention. To illustrate this portability, $B_{Y}$ was applied to published confusion matrices from an independent diabetes progression study in a clinically distinct setting where two sequential classifiers operate under markedly different prevalence regimes. The classical Youden index ranked both classifiers as nearly equivalent and inverted their relative ordering; $B_{Y}$ correctly identified the low-prevalence classifier as substantially superior, with corrections from $C_{Y}$ to $B_{Y}$ proportional to the degree of imbalance in each task. This external illustration confirms that the sensitivity of $B_{Y}$ to prevalence is a structural property of the index that generalizes to any binary classification context where the distribution of classes carries meaningful information about real-world performance.

The uncertainty associated with all reported point estimates has been quantified through a parametric bootstrap procedure grounded in the binomial sampling model of the confusion matrix. Since sensitivity and specificity are maximum likelihood estimators of binomial proportions, their sampling variability propagates analytically to $B_{Y}$, its maximum, and the intersection points $P_{1}$ and $P_{2}$ through B = 2,000 bootstrap replications. For the HOMA-IR classifier the 95% confidence intervals are: $C_{Y}=0.890$ [0.782, 0.978]; *max* $B_{Y}$  = 0.913 [0.823, 0.997]; *P* at *max* $B_{Y}=0.334$ [0.027, 0.575]; $P_{1}=0.190$ [0.118, 0.542]; $P_{2}=0.518$ [0.504, 0.652]. The relatively wide interval for the prevalence at which $B_{Y}$ is maximized reflects the geometric flatness of the $B_{Y}$ curve near its peak when sensitivity and specificity are both high and close to each other, a property of the classifier rather than a limitation of the index. That $P_{2}$ was recoverable in only 1,082 of 2,000 replications is itself informative: it indicates that under sampling variability the second intersection is not always guaranteed, which quantifies the instability of this boundary and should be taken into account when using $P_{2}$ as a deployment threshold. The complete bootstrap procedure, including a dedicated Robustness Check module that reports the empirical bootstrap distributions, their skewness, percentile and BCa confidence intervals, and a non-parametric case-resampling comparison, is implemented in the interactive Shiny application accompanying this manuscript, where any user can enter their own confusion matrix and reproduce the full uncertainty and robustness analysis directly.

Finally, the eigenvalue analysis of the confusion matrix reveals that the relationship between $B_{Y}$ and $C_{Y}$ is not merely analogical but mathematically grounded in the underlying structure of binary classification. The trace and determinant of the confusion matrix, functions of its eigenvalues, encode the same prevalence-dependent information that $B_{Y}$ captures through *PPV* and *NPV*, providing an algebraic foundation for the proposed extension that connects it to the spectral properties of the classification operator itself. This connection suggests that $B_{Y}$ is not an *ad hoc* modification of an existing index but a natural consequence of applying Bayes' theorem to the confusion matrix, and it opens a principled path toward further extensions in multi-class settings where the spectral structure of larger confusion matrices may yield analogous prevalence-sensitive generalizations.

## Conclusions

In this work, the epistemological equivalence and clinical utility of sensitivity and specificity from a Bayesian perspective have been demonstrated. These correspond respectively to the Positive Predictive Value and Negative Predictive Value. This distinction is not trivial: while the classical approach informs about classifier performance under controlled conditions by fixing the disease status, the Bayesian approach answers the clinician's fundamental question, given that a surrogate index yielded a positive result, what is the actual probability that the patient has insulin resistance? The use of Bayesian metrics allows for a direct interpretation of medical uncertainty that the frequentist approach conceals within the likelihood ratio framework.

The Bayesian Youden Index, proposed here as a prevalence-dependent extension of the classical Youden index, provides a principled framework for evaluating a classifier's ability to correctly segregate populations as a function of the real-world class distribution. The mathematical equivalence between Bayesian sensitivity and specificity and the predictive values, together with the eigenvalue structure of the confusion matrix, demonstrates that this extension is not merely analogical but is grounded in the underlying algebra of binary classification. This integrated framework enables direct comparison of classification performance across surrogate insulin resistance indices through both classical and Bayesian lenses. Although developed and validated within the insulin resistance domain, the index requires only the four cells of any confusion matrix and the class prevalence, quantities universally available in any binary classification problem, which makes it applicable to any clinical or algorithmic context without modification.

Regarding class imbalance, prevalence can be used as a natural measure of group asymmetry. As demonstrated both analytically and through cross-domain illustration, although the behavior of the Bayesian Youden index is not symmetric around P=0.5, a particular prevalence regime associated with class imbalance can yield a Bayesian Youden value superior to the classical index. The intersection points $P_{1}$ and $P_{2}$ identify the exact prevalence thresholds at which the two frameworks agree, providing practitioners with actionable decision boundaries rather than a single aggregate number. This is of particular relevance for those working with imbalanced groups: without first studying whether the imbalance is compensated by the obtained sensitivity and specificity values, a classifier should neither be dismissed prematurely nor subjected to data-level balancing techniques whose additional complexity and assumptions may not be warranted.

Finally, uncertainty in all reported point estimates: the classical Youden index, the maximum Bayesian Youden index, the prevalence at which it is attained, and the intersection points $P_{1}$ and $P_{2}$ has been quantified through a parametric bootstrap procedure grounded in the binomial sampling model of the confusion matrix. Bootstrap 95% confidence intervals confirm that the key statistics are estimable with meaningful precision given the available sample, and the accompanying interactive Shiny application allows any user to apply the same uncertainty quantification to their own classifier by entering their confusion matrix directly.

## Data Availability

The original contributions presented in the study are included in the article/Supplementary Material, further inquiries can be directed to the corresponding author.
